# Zebrafish as a Model for Cardiovascular Disease Using Nanotechnology and Emerging Optogenetic Tools

**DOI:** 10.3390/biomedicines14030596

**Published:** 2026-03-07

**Authors:** Phuc Nguyen, Vanessa Avila, Juhyun Lee

**Affiliations:** 1 Department of Bioengineering, The University of Texas at Arlington, Arlington, TX 76010, USA; pxn9757@mavs.uta.edu (P.N.); vanessa.avila@uta.edu (V.A.); 2 Department of Electrical Engineering and Computer Science, Daegu Gyeongbuk Institute of Science and Technology (DGIST), Daegu 42988, Republic of Korea

**Keywords:** zebrafish, nanoparticle, optogenetics

## Abstract

Recent advances in experimental model systems have improved our ability to study cardiovascular development, function, and disease with high spatial and temporal resolution. The zebrafish (*Danio rerio*) has emerged as a powerful vertebrate model for cardiovascular research due to its transparency, genetic tractability, and conserved cardiac physiology, similar to humans. These features allow real-time in vivo imaging, the functional assessment of cardiac performance, and the tracking of signaling pathways that are fundamental in cardiovascular development and disease. Recent advances in nanotechnology and optogenetics have introduced complementary tools for probing and manipulating cardiovascular systems with high spatial and temporal precision. Nanoparticle-based platforms enable the tunable delivery of drugs, nucleic acids, and imaging agents, while optogenetic systems allow the light-mediated control of gene expression, signaling pathways, and cardiac electrophysiology. In this review, we summarize recent progress in the application of nanoparticle-based technologies and the emerging optogenetic tools in zebrafish cardiovascular research, including the optical control of cardiac signaling and electrophysiology. We briefly discuss emerging complementary efforts toward nanoparticle and optogenetic approaches, how to overcome key technical limitations, such as light penetration and gene delivery, and how to facilitate the development of fully optical platforms for cardiovascular disease modeling and drug screening.

## 1. Introduction

Cardiovascular diseases (CVDs) remain one of the leading causes of mortality worldwide. According to the American Heart Association, in the United States, CVD accounts for 915,973 deaths, or 1 death every 34 s [1,2]. These disorders encompass a broad spectrum of pathologies, including hypertension, stroke, myocardial infarction, arterial stiffness, and vascular dysfunction [3]. Despite advances in diagnosis and treatment, the complex molecular and biomechanical mechanisms underlying CVD progression remain incompletely understood, underscoring the need for physiologically relevant and experimentally tractable model systems.

In recent years, the zebrafish (*Danio rerio*) has emerged as a powerful vertebrate model for studying cardiovascular development, function, and disease. Zebrafish offer several distinct advantages, including optical transparency during early development, rapid embryogenesis, a conserved cardiac structure, and genetic manipulation [4]. These features enable real-time imaging, the quantitative assessment of cardiac physiology, and the interrogation of conserved signaling pathways that govern cardiovascular development and disease.

Concurrently, nanoparticle-based approaches and optogenetic tools have become increasingly popular techniques as advanced technologies for probing cardiovascular mechanisms and manipulating cellular behavior [5,6,7,8,9]. Nanoparticles enable the targeted delivery of nucleic acids and imaging agents, while optogenetic systems provide a precise spatiotemporal control of gene expression, signaling pathways, and cellular activity [5,10]. By facilitating these technologies into the zebrafish model, we can investigate cardiovascular disease mechanisms at both the genetic and tissue levels, thereby enhancing mechanistic insight and enabling the development of more targeted therapeutic strategies [6].

Furthermore, the zebrafish offers a range of experimentally accessible methods for delivering nanoparticles and optogenetic agents across different developmental stages, enabling flexible integration into cardiovascular research workflows. During early embryogenesis, microinjection at the one-cell stage or into specific anatomical sites, such as the yolk sac, pericardial space, or circulation, provides precise control over dosage and location [11]. These techniques are commonly employed in nanoparticle biodistribution studies, toxicity evaluations, and nucleic acid delivery, including the introduction of optogenetic constructs via plasmid DNA or mRNA to produce either transient or widespread expression [12]. In larval and adult zebrafish, intravascular or intramyocardial microinjections facilitate the direct delivery of nanoparticles, lipid carriers, or genetic material into the cardiovascular system under physiological flow conditions, enabling studies on circulation, vascular targeting, and cardiac function [13]. Additionally, bath immersion offers a straightforward, scalable method for systemic exposure to nanoparticles or small molecules, supporting the high-throughput screening of cardiovascular effects and toxicity, though with less tissue specificity [11,14]. Genetic techniques, such as transient expression via injected nucleic acids and the use of transgenic lines, enable the stable or cell-specific expression of optogenetic actuators in cardiac and vascular tissues [7,15].

This review summarizes recent and emerging approaches in the use of zebrafish models to study cardiovascular disease, emphasizing the integration of biological relevance with emerging technological innovations. We focus specifically on the application of nanoparticle-based technologies and optogenetic tools in zebrafish, highlighting how these approaches are accelerating the discovery of disease mechanisms and facilitating translational progress toward novel cardiovascular therapies. We further provide a comparative overview of the key advantages and limitations of the major nanoparticle and optogenetic strategies discussed in Table 1, underscoring both their current capabilities and remaining technical challenges.

## 2. Nanoparticle-Mediated Delivery of Therapeutics and Gene Editors in Zebrafish Cardiovascular Models

### 2.1. Polymeric Nanoparticles as Tunable Cardiovascular Delivery Platforms

Polymeric nanoparticles are among the most widely used nanocarriers in zebrafish cardiovascular research due to their biocompatibility, structural versatility, and capacity for controlled drug release. Commonly used systems include poly(lactic-co-glycolic acid) (PLGA), chitosan, and polyethylene glycol (PEG)-based nanoparticles [16,17,18]. PLGA is particularly attractive for zebrafish cardiomyocyte studies because of its predictable hydrolytic degradation into lactic and glycolic acids, which are naturally metabolized through the tricarboxylic acid cycle and exhibit minimal cardiac toxicity. This favorable biodegradation profile has supported regulatory approval by both the FDA and EMA for clinical applications, including drug delivery and intravascular devices, facilitating translational relevance from zebrafish models to mammalian systems. Importantly, the physicochemical properties of PLGA nanoparticles, such as particle size, surface charge, polymer composition, and degradation rate, can be precisely tailored, enabling the sustained and controlled delivery of cardiovascular therapeutics. In zebrafish cardiomyocytes, adjusting particle size and surface charge affects how particles penetrate the heart tissue, enter cells, and are cleared. Modifying the lactic-to-glycolic acid ratio provides precise control over the rate at which these particles degrade and release drugs [19]. For example, PLGA nanoparticles were designed to carry an NICD plasmid and tested as a non-viral gene delivery method in developing zebrafish [19]. Toxicity assessments revealed that PLGA at doses up to 50 µg/mL did not significantly impact survival or cause major deformities compared to controls, suggesting good biocompatibility [19]. NICD-loaded PLGA nanoparticles conjugated with Tie2/Tie1 antibodies were injected into 48 hpf larvae via the bloodstream [19]. They circulated, targeted the endocardium, and activated Notch-related genes. This activation improved cardiac function, shown by increased ejection fraction and cardiac output, confirming safe and effective in vivo plasmid delivery in the zebrafish model [19]. These features support the sustained and targeted delivery of cardiovascular treatments, including growth factors, small molecules, nucleic acids, and gene-editing cargos, during development, injury, and regeneration.

### 2.2. Chitosan Nanoparticles for Gene and siRNA Delivery

**Figure 1:**
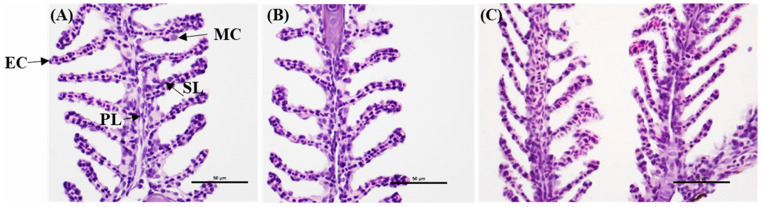
Histological evaluation of zebrafish gill tissue following chitosan nanoparticle and miR-155-CNP administration. Representative hematoxylin and eosin (H&E)-stained longitudinal sections of adult zebrafish gill filaments at 48 h post-administration. (**A**) Control (H_2_O-injected) fish displaying normal gill morphology, including epithelial cells (ECs), primary lamellae (PL), secondary lamellae (SL), and mucous cells (MCs). (**B**) Chitosan nanoparticle (CNP)-injected fish showing preserved lamellar structure without observable pathological alterations. (**C**) miR-155-loaded chitosan nanoparticle (miR-155-CNP)-injected fish, demonstrating intact epithelial organization and normal secondary lamellar architecture comparable to control. No evidence of epithelial lifting, lamellar fusion, edema, or inflammatory infiltration was observed. Scale bars = 50 μm [22].

Chitosan nanoparticles function as effective vehicles for nucleic acid delivery in zebrafish models of cardiomyocyte disease due to their strong cationic properties and favorable interactions with cardiac cells [20,21]. Protonated amino groups on chitosan electrostatically bind negatively charged DNA and RNA, forming stable nano-polyplexes that protect nucleic acids from enzymatic degradation in physiological environments [21]. The strong electrostatic interactions of chitosan nanoparticles allow them to effectively compete with serum proteins in zebrafish plasma, preserving cargo integrity and enhancing myocardial bioavailability. This delivery strategy was demonstrated in vivo in zebrafish using chitosan nanoparticles encapsulating dre-miR-155 mimics [22]. Following intraperitoneal administration, these nanoparticles achieved efficient tissue-level overexpression, significantly reduced viral burden, and improved survival during viral hemorrhagic septicemia virus (VHSV) infection. The functional data demonstrate that miR-155 delivery is efficient, leading to immune gene modulation, improved survival, and lower viral loads, directly supporting the histological findings shown in (Figure 1). Despite achieving effective systemic gene regulation, H&E staining reveals that the gill architecture remains intact without signs of inflammation or structural damage [22]. Overall, these results demonstrate that chitosan nanoparticles enable effective nucleic acid delivery and functional gene regulation in zebrafish cardiovascular-relevant tissues while preserving tissue biocompatibility and safety. Additionally, chitosan nanoparticles promote cellular uptake and facilitate endosomal escape through the proton-sponge effect, which is essential for successful cytoplasmic release and gene silencing [23]. These properties make chitosan-based systems particularly useful for delivering siRNA and gene-regulatory molecules to cardiovascular tissues in zebrafish. However, excessive surface positive charge can cause issues such as non-specific cellular uptake, unintended interactions with intracellular membranes, and off-target accumulation, including possible mitochondrial association [24]. These effects may lead to mitochondrial membrane disruption, oxidative stress, and cytotoxicity. Moreover, highly cationic surfaces are linked to increased immunogenicity and inflammation, especially at higher doses or with long-term exposure. To mitigate these concerns, strategies like surface modification with neutral or hydrophilic polymers (PEGylation), partial charge shielding, charge-tunable formulations, and careful dose optimization are used to balance transfection efficiency with biocompatibility [25]. Adding targeting ligands or stimuli-responsive components can further reduce non-specific uptake and improve delivery specificity.

### 2.3. Nanoparticle-Mediated Gene Editing and Cardiovascular Regulation

Nanoparticles serve as powerful vectors for gene modulation and genome editing in the zebrafish cardiovascular disease model, including the delivery of CRISPR/Cas9 components and siRNA constructs that influence pathways involved in hypertension, vascular stiffness, and cardiac hypertrophy [26,27,28]. For example, CRISPR-Cas9-mediated gene knockouts in zebrafish were generated by microinjecting sgRNA and Cas9 mRNA into one-cell embryos, achieving 60–100% indel efficiency, as confirmed by PCR, T7EI assay, and sequencing, and enabling the establishment of stable homozygous mutant lines [29]. Crossing these mutants with Tg(fli1a:eGFP) reporter fish revealed markedly reduced vessel length, density, and branching by live imaging [29]. Also, these vascular and metabolic perturbations directly alter cardiomyocyte mechanical load, calcium handling, and hypertrophic signaling, making gene-targeted nanoparticle delivery a valuable tool for dissecting the mechanisms of cardiomyocyte disease [30,31,32]. For example, the nanoparticle-mediated targeting of disease-associated genes such as PCSK9, ANGPTL3, and TTR directly influences lipid metabolism and vascular stiffness [26]. The delivery of CRISPR/Cas9 target angiotensinogen (AGT) in macrophages and endothelial cells sustained reductions in both systolic and diastolic blood pressure, directly linking nanoparticle-based gene editing to hypertension management [27,28]. Furthermore, the CRISPR-mediated disruption of G6PD and GPER1improves arterial elasticity, decreases vascular stiffness, and normalizes vascular tone, which are key factors in hypertension-related vascular remodeling [28]. The nanoparticle-assisted targeting of PCSK9 and APOC3 further reduces lipid accumulation, macrophage infiltration, and vascular inflammation, consequently alleviating the hemodynamic stress that contributes to cardiac hypertrophy [28].

### 2.4. Lipid-Based Nanoparticles for Cardiovascular Therapeutics and Toxicity Models

Lipid-based nanoparticles, such as liposomes, solid lipid nanoparticles (SLNs), and mRNA lipid nanoparticles (LNPs), are routinely used to deliver chemotherapeutics, antioxidants, or RNA molecules into zebrafish cardiovascular tissues [33,34,35]. Their lipid bilayer or lipid matrix closely mimics biological membranes, enabling the efficient encapsulation and intracellular delivery of hydrophobic drugs, siRNA, and mRNA while protecting cargos from enzymatic degradation [33]. In zebrafish research, liposomal formulations of doxorubicin serve as a primary tool for modeling chemotherapy-induced cardiomyopathy, enabling researchers to observe heart wall thinning and contractile failure in transgenic reporter lines such as cmlc2:GFP or tg(flk1:mCherry) [33]. In addition, LNPs deliver modified mRNA into the myocardium, resulting in robust luciferase and eGFP expression in heart tissue 24 h after intramyocardial administration [34]. Additionally, when microinjected post-fertilization into regions such as the hindbrain ventricle, trunk, caudal vein, or pericardial cavity, LNP-encapsulated mRNA induced strong, tissue-specific GFP expression in the brain, skeletal muscle, blood vessels, and heart [36]. Conversely, naked mRNA showed minimal expression. The embryos maintained high survival rates with no indications of toxicity. These results demonstrate that LNPs enable efficient, targeted, and safe mRNA delivery in zebrafish. Furthermore, solid lipid nanoparticles (SLNs) enhance the bioavailability and reduce the systemic toxicity of chemotherapeutic and antioxidant compounds, such as doxorubicin, paclitaxel, and methotrexate [35].

## 3. Targeting Cardiovascular Tissues via Surface-Engineered Nanoparticles

### 3.1. Surface Modification and Targeted Cardiovascular Nanoparticles

Beyond core composition, surface engineering is a key determinant of nanoparticle behavior in the zebrafish cardiovascular system, where rapid circulation, a developing immune environment, and endothelial barriers strongly influence myocardial delivery [37]. PLGA nanoparticles can be functionalized with PEG to improve colloidal stability and prolong circulation time, while reactive groups, such as maleimide, enable the conjugation of targeting ligands [17]. For instance, PLGA–PEG–maleimide formulations allow the attachment of targeting peptides such as S2P, conferring cardiac or endothelial specificity and improving localization within cardiovascular tissues [18]. PEGylation also minimizes nonspecific protein adsorption and reduces uptake by innate immune cells, such as macrophages and neutrophils, which are readily visualized in transgenic zebrafish lines [17]. For example, silica nanoparticles (SiNPs) encapsulating RhB, with or without PEG surface modification, were prepared and microinjected into 2-day-old transgenic zebrafish to evaluate vascular toxicity [38]. In Tg(mpo:GFP) zebrafish, unmodified SiNPs significantly increased neutrophil recruitment, signaling systemic inflammation, while PEG-modified SiNPs greatly lessened this inflammatory response [38]. In Tg(fli-1:EGFP) fish, SiNPs hindered sub-intestinal vessel (SIV) growth and caused endothelial damage, but PEGylation notably lowered the rate of angiogenesis inhibition [38]. Complementary in vitro tests on endothelial cells revealed that PEG modification decreased ROS production, mitochondrial damage, apoptosis, and the expression of pro-inflammatory cytokines. These surface-engineered platforms offer a flexible approach to improving targeting efficiency while minimizing off-target effects in zebrafish cardiovascular models.

### 3.2. Hemodynamics-Dependent Nanoparticle Transport and Cardiovascular Toxicity

The zebrafish circulatory system provides a physiologically relevant platform for studying nanoparticle transport under realistic hemodynamic conditions [39,40,41]. Exposure to metal, polymer, and carbon-based nanoparticles induced characteristic cardiovascular phenotypes, including pericardial edema, bradycardia, cardiac arrhythmias, and reductions in stroke volume, ejection fraction, and cardiac output, making these parameters sensitive indicators of cardiotoxicity [39]. These functional impairments are often accompanied by vascular leakage and increased endothelial permeability, reflecting compromised vascular integrity and the dose-dependent disruption of cardiac rhythm and contractile performance [42]. Importantly, zebrafish models enable a direct investigation of how hemodynamic factors influence nanoparticle transport, margination, and vascular interaction [40,41]. Blood flow within zebrafish vasculature generates heterogeneous shear stress profiles, including low, oscillatory, and high shear, similar to those observed in human arteries, bifurcations, and stenotic regions [40]. Low and disturbed shear stress promotes nanoparticle margination toward the vessel wall and enhances endothelial uptake, whereas high shear stress reduces residence time and cellular interaction, directly linking hemodynamics to nanoparticle distribution and targeting efficiency [41]. Computational and experimental studies using a hybrid nanoparticle, such as the Ag/Al_2_O_3_ system under stenotic flow conditions, further demonstrate that shear-dependent changes in velocity profiles and residence times lead to preferential accumulation in low-shear and disturbed-flow regions [41]. Comparing low (LSS), oscillatory (OSS), and high shear stress (HSS) conditions shows that LSS and OSS significantly boost endothelial NP uptake [41]. This increase is partly driven by higher ROS production, decreased glycocalyx integrity, increased membrane fluidity, and the upregulation of clathrin- and caveolin-mediated endocytosis pathways [41]. Conversely, HSS reduces NP internalization because of its shorter contact time with endothelial cells. Zebrafish imaging further reveals that nanoparticles tend to accumulate more in low-velocity venous regions rather than in high-flow arterial vessels, connecting flow dynamics with vascular distribution and potential toxicity [41]. Together, these findings highlight the importance of hemodynamic context in the design of nanoparticles for cardiovascular targeting and safety assessment.

### 3.3. Biomimetic Nanoparticles for Targeting Injured Myocardium and Inflamed Vasculature

**Figure 2:**
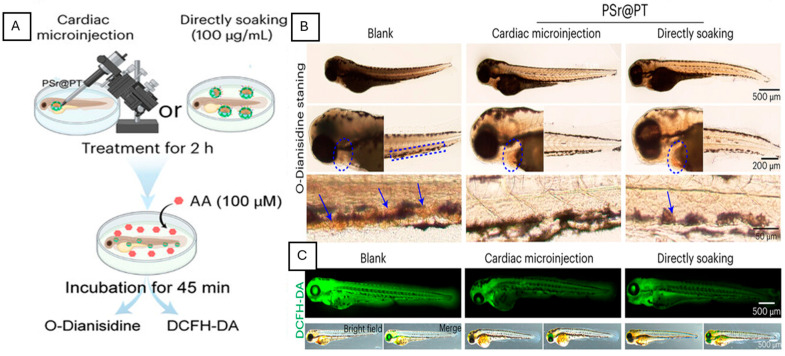
PSr@PT biomimetic nanomotors inhibit thrombosis and oxidative stress in zebrafish. (**A**) Experimental schematic showing PSr@PT nanoparticle delivery by cardiac microinjection or direct soaking (100 µg mL^−1^) for 2 h, followed by arachidonic acid (AA, 100 µM)-induced thrombosis and staining with O-dianisidine and DCFH-DA. (**B**) O-dianisidine staining of zebrafish larvae demonstrates that arachidonic acid (AA) induces pronounced thrombus formation in the caudal vasculature (blue arrows), while red blood cells (RBCs) within the heart are indicated by blue circles. Administration of PSr@PT nanoparticles significantly reduces AA-induced caudal venous thrombus formation compared to untreated controls. (**C**) DCFH-DA fluorescence images showing reduced ROS levels in PSr@PT-treated larvae compared to blank controls. Scale bars: 500 µm, 200 µm, and 50 µm [46].

Biomimetic nanoparticles, including cell-membrane-coated particles or peptide-functionalized carriers, are designed to mimic natural biological interactions and thereby enhance the repair of injured zebrafish myocardium or the treatment of inflamed endocardium [43,44,45]. Cell membrane-coated nanoparticles retain native adhesion proteins such as P-selectin ligands, enabling selective binding to damaged endothelium, atherosclerotic plaques, and thrombotic sites while simultaneously evading immune clearance [43]. This targeting strategy was experimentally validated using platelet–thylakoid hybrid membrane-coated polydopamine nanomotors that preserve platelet membrane proteins, including CD47 and glycoprotein receptors [46]. In zebrafish, these nanoparticles were introduced by cardiac microinjection (5 nL per larva) or by direct soaking (100 μg·mL^−1^) at 72 h post-fertilization, enabling systemic vascular exposure without invasive surgery [46]. Following arachidonic acid-induced thrombosis, treated zebrafish exhibited a marked reduction in caudal vein thrombus area, restored erythrocyte flow, and significantly lower ROS levels than untreated controls, demonstrating the effective suppression of early thrombus formation through platelet inhibition and the modulation of oxidative stress (Figure 2) [46]. Another application of nanoparticles involves incorporating or mimicking natural cell-surface components, specifically by targeting the CD47–SIRPα signaling pathway, an endogenous cell–cell recognition pathway used by healthy cells to regulate immune interactions [43,44]. By mimicking native CD47 ligands, nanoparticles selectively accumulate in inflamed tissues characterized by high macrophage activity, conditions that closely resemble injured myocardium and inflamed vasculature [44]. Furthermore, mesoporous silica nanoparticles coated with human ventricular cardiomyocyte membranes maintain native membrane proteins, including cadherins and homing receptors, which facilitate ligand–receptor recognition with cardiac cells [45]. As a result, these biomimetic nanoparticles showed significantly increased uptake by cardiomyocytes (~92%) compared to uncoated nanoparticles (~37%), while reducing uptake by non-cardiac cell types, such as lung and liver cell lines [45]. In parallel, peptide-functionalized biomimetic carriers target markers overexpressed during zebrafish cardiac repair, such as collagen types (col1a1a) and icam1, resulting in superior localization to the injured apex [47]. These carriers achieve significantly higher payload accumulation than non-biomimetic variants, a process that can be tracked in real time across the optically transparent larval stages.

## 4. Zebrafish as a Platform for Studying Nanoparticle Responsiveness and Cardiovascular Imaging

### 4.1. PEGylation-Dependent Circulation Dynamics in Zebrafish Vasculature

**Figure 3:**
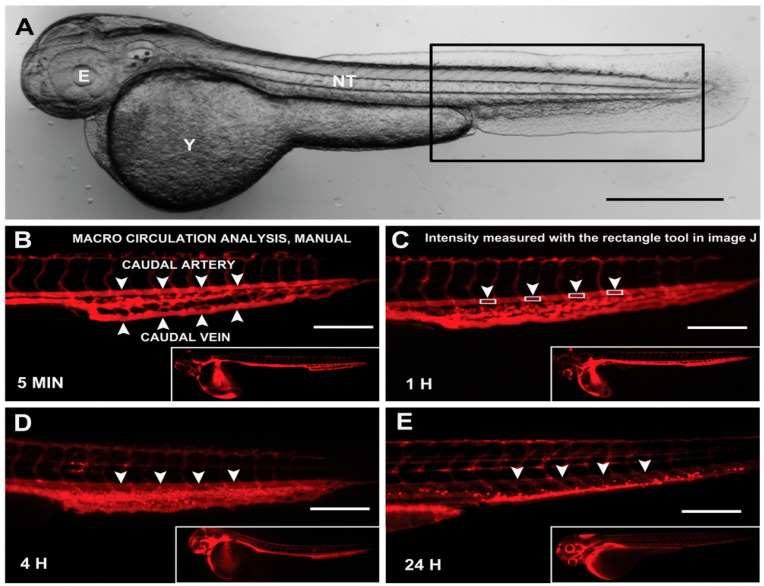
**PEGylation-dependent nanoparticle circulation in zebrafish vasculature.** Manual analysis of nanoparticle circulation was performed in 48 h post-fertilization zebrafish embryos. (**A**) Transmission image of the whole embryo, with the rectangular inset indicating the caudal region selected for imaging. (**B**–**E**) Fluorescent images of embryos injected with 100 nm PEGylated liposomes acquired at (**B**) 5 min, (**C**) 1 h, (**D**) 4 h, and (**E**) 24 h post-injection. Major vascular structures, including the caudal artery and caudal vein (arrowheads), are identified. Rectangular regions used for fluorescence quantification were positioned on the caudal artery and between intersegmental vessels. A progressive reduction in arterial fluorescence is observed at 4 and 24 h. Fluorescence intensity values were normalized to the total nanoparticle signal in the whole embryo. Scale bars: 500 μm (**A**) and 200 μm (**B**–**E**) [48].

PEGylation has been shown to significantly influence nanoparticle circulation and clearance in the zebrafish cardiovascular system [48]. For example, fluorescent nanoparticles with varying surface chemistries, including PEGylated and non-PEGylated liposomes, were injected into 2 dpf zebrafish embryos via IV to assess circulation time and cellular uptake through automated image analysis [48]. Circulation was tracked by monitoring fluorescence in the caudal artery over 72 h and normalized to the whole-embryo signal. PEGylated nanoparticles, especially 100 nm liposomes, showed extended vascular circulation, detectable for up to 72 h, while non-PEGylated liposomes were cleared within hours (Figure 3) [48]. Further analysis with transgenic reporter lines indicated that rapid clearance was associated with higher uptake by macrophages and endothelial cells, whereas PEGylation decreased cellular uptake and prolonged circulation [48]. PEG surface coatings markedly reduce uptake by immune and endothelial cells, which directly correlate with extended vascular circulation times and improved stability. Notably, the circulation behavior of PEGylated nanoparticles in zebrafish closely resembles that in murine models, confirming the translational relevance of zebrafish for studying nanoparticle pharmacokinetics and biodistribution [47,48]. By reducing protein adsorption and cellular internalization, PEGylation slows nanoparticle clearance and extends exposure within the vasculature [48].

### 4.2. Stimuli-Responsive Nanoparticles for Disease-Specific Cardiovascular Targeting

Stimuli-responsive nanoparticles offer a sophisticated layer of precision by releasing therapeutic payloads in response to disease-specific microenvironmental cues. In the context of zebrafish cardiomyocyte injury, these cues include localized acidosis (pH), elevated reactive oxygen species (ROS), and altered shear stress within the narrowed or regenerating endocardial lumen [33,34,35]. These nanoparticle-based drug delivery systems (NP-NDDSs) are engineered to sense pathological conditions and enable spatiotemporal control of therapeutic release [49]. For example, folate-functionalized chitosan/rGO/NiO nanocomposites serve as pH-sensitive drug carriers and have been tested for biocompatibility in zebrafish embryos [50]. They release doxorubicin at pH 5.5, simulating diseased tissue conditions, with approximately 98% release, whereas release is minimal at pH 7.4 [50]. As a result, folate enhances cellular uptake and cytotoxicity by targeting specific receptors. In vivo safety was evaluated by microinjecting 10 nL of suspension into embryos and monitoring them for 72 h [50]. Free NiO caused mild effects but incorporating it into the CS/rGO matrix improved biocompatibility, resulting in over 90% hatching rates and no significant issues [50]. In zebrafish models of cryoinjury or genetic cardiomyopathy, ischemic myocardium and inflamed vasculature exhibit distinct physicochemical features, such as acidosis, excessive ROS production, hypoxia, and altered shear stress, making them ideal targets for stimulus-responsive delivery strategies [51]. By responding selectively to these cues, stimuli-responsive nanocarriers are preferentially accumulated in inflamed myocardium and activated vasculature, where they can modulate immune responses, including neutrophil phenotypic switching, macrophage polarization, and lymphocyte balance [49]. This target release mechanism overcomes key limitations of conventional systemic anti-inflammatory therapies by reducing off-target exposure and enhancing therapeutic efficacy [49]. Consequently, stimuli-responsive nanoparticles represent a highly relevant and forward-looking platform for modeling human cardiovascular disease and testing precision nanomedicine strategies in zebrafish models.

### 4.3. Carbon-Based Nanomaterials as Models of Vascular Inflammation and Fibrotic Remodeling

**Figure 4:**
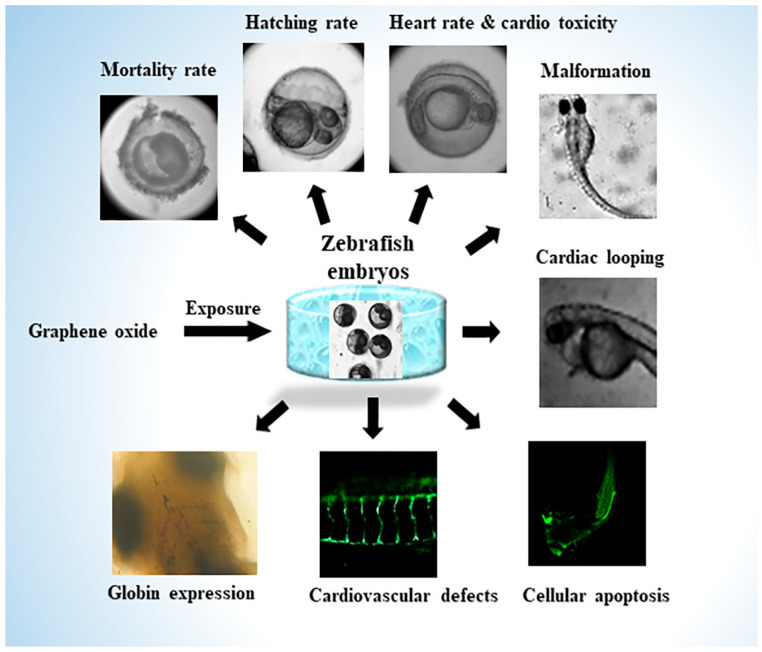
Graphene oxide-induced developmental and cardiovascular toxicity. Overview of the developmental and cardiovascular toxicity induced by graphene oxide (GO) in zebrafish embryos. Exposure to GO leads to several adverse effects, including reduced hatching rates, increased mortality, cardiac malformations, impaired cardiac looping, altered heart rate, disrupted globin expression, endothelial dysfunction, cardiovascular defects, and apoptosis [55].

Carbon-based nanomaterials, including graphene oxide (GO) and carbon nanotubes (CNTs), have been widely employed in zebrafish models to interrogate mechanisms of cardiomyocyte disease, particularly those driven by vascular inflammation, oxidative stress, and fibrotic remodeling [52,53,54]. In zebrafish larvae, GO nanoparticles accumulate within cardiovascular tissues, including the heart and tail vasculature, enabling direct interaction with endothelial cells and circulating immune cells (Figure 4) [52,55]. To be specific, GO was used to model cardiovascular inflammation and fibrotic-like changes in zebrafish embryos by exposing fertilized eggs (from 5 to 120 h post-fertilization) to increasing GO concentrations (0.1–1 mg/mL) [55]. A dose-dependent toxicity was evident, including higher mortality rates, delayed hatching, increased heart rate, and notable morphological issues such as pericardial edema, cardiac looping defects (evidenced by increased SV–BA distance), and shorter body length [55]. In transgenic Tg(fli1a:EGFP) larvae, GO disrupted blood vessel development, leading to irregular intersegmental vessel branching and impaired angiogenesis. Additionally, GO exposure caused apoptosis, reduced hemoglobinization, and indicated compromised hematopoiesis and vascular function [55]. This accumulation is associated with pronounced oxidative stress, as evidenced by the increased expression of antioxidant markers such as total superoxide dismutase (T-SOD) and inducible nitric oxide synthase (iNOS), reflecting the disruption of vascular redox homeostasis [52]. At higher concentrations, GO exposure causes pericardial edema, altered blood flow velocity, compromised endothelial barriers, and the activation of apoptosis-related genes, a hallmark feature of endothelial injury and early fibrotic remodeling [52]. These pathological effects are closely linked to ROS production, oxidative DNA damage, and apoptosis in vascular tissues, confirming that the inherent reactivity of carbon nanomaterials makes them useful tools for studying vascular inflammation and fibrosis-related cardiovascular toxicity [54]. Similarly, CNT exposure in zebrafish embryo results in pericardial edema, vascular hyperemia, and compromised endothelial barriers [53]. Molecular analyses reveal dose-dependent increases in oxidative stress markers, including 8-hydroxy-2′-deoxyguanosine (8-OHdG) and nucleolar stress marker NOP10, indicating oxidative DNA damage and cellular stress response [53]. Notably, CNT-based nanomaterials caused more severe effects than GO, likely due to their tubular geometry, smaller size, and enhanced cellular penetration, leading to increased ROS production and inflammatory signaling [53].

### 4.4. Iron Oxide and Redox-Active Nanoparticles for Oxidative Stress Assessment

Iron oxide nanoparticles (FeNPs) possess superparamagnetic properties, making them effective MRI contrast agents for the non-invasive visualization of vascular structures in zebrafish models of cardiomyocyte disease [56]. In addition to imaging capability, FeNPs exhibit significant redox activity, demonstrating high free-radical-scavenging capacity across various oxidative stress tests, including DPPH, ABTS, nitric oxide, hydrogen peroxide, and FRAP, with approximately 90% inhibition at assessing FeNP-induced oxidative effects, as nanoparticle accumulation, oxidative stress responses, toxicity, morphology, behavior, and survival can be evaluated concurrently [57]. For example, green-synthesized iron oxide (ferric) nanoparticles (FeNPs), derived from Madhuca indica, were characterized and tested for antioxidant ability and in vivo toxicity using zebrafish embryos [57]. Antioxidant tests (DPPH, FRAP, ABTS, H_2_O_2_, and NO) showed strong redox activity, with about 90–94% radical scavenging at 50 µg/mL, indicating effective ROS-neutralizing ability [57]. Also, at lower concentrations (5–10 µg/mL), all embryos survived and hatched normally, showing good biocompatibility [57]. Higher doses (≥40 µg/mL), however, greatly decreased hatching and survival rates and caused dose-dependent toxicity, consistent with oxidative stress [57]. These results show that redox-active FeNPs have strong antioxidant effects at controlled doses but may induce oxidative stress-related developmental toxicity at higher levels in zebrafish. Importantly, FeNPs, along with other inorganic nanoparticles such as gold and silver, can modulate reactive oxygen species (ROS) levels via surface redox reactions. These ROS-mediated processes are closely linked to endothelial dysfunction and inflammatory signaling, including the activation of NF-κB and MAPK pathways, thereby connecting nanoparticle exposure to vascular inflammation and cardiovascular pathology [58]. Altogether, redox-active nanoparticles serve not only as imaging agents but also as functional tools for probing oxidative stress-driven mechanisms underlying cardiovascular disease in zebrafish models [56].

### 4.5. Nanoparticles as Imaging Agents in Zebrafish Cardiovascular Models

Zebrafish embryos offer unique advantages for nanoparticle-based cardiovascular imaging due to their optical transparency and rapid development [48]. These features allow the real-time, non-invasive imaging of nanoparticle circulation through the beating heart, microvasculature, and developing tissues, which is not feasible in mammalian models. For example, fluorescent silica nanoparticles were used as vascular imaging agents in transgenic zebrafish embryos to evaluate real-time cardiovascular distribution and biocompatibility [59]. The nanoparticles were injected intravenously, and high-resolution fluorescence microscopy enabled the tracking of blood flow dynamics, endothelial interactions, and tissue accumulation within the transparent zebrafish vasculature [59]. The particles produced strong, stable fluorescence signals that allowed the visualization of subintestinal vessels and microvascular networks without significantly affecting survival or causing major developmental abnormalities at controlled doses [59]. Zebrafish share conserved genetic and physiological pathways with humans, including Notch, VEGF, renin–angiotensin signaling, inflammatory cascades, and oxidative stress responses [60,61,62]. Inorganic nanoparticles, such as gold, silver, iron oxide, quantum dots, and silica particles, possess unique optical and magnetic properties that facilitate imaging of vascular structures, tracking nanoparticle biodistribution, and evaluating oxidative stress or inflammatory responses [56,57,58]. In fact, gold and silver nanoparticles exhibit strong surface plasmon resonance, enabling the high-contrast optical imaging of blood vessels and real-time tracking of nanoparticle distribution in vivo [56].

## 5. Optogenetic Control of Cardiac Function in Zebrafish Models

Optogenetic tools have gained significant popularity in cellular manipulation through the use of light-sensitive proteins. Zebrafish represent an ideal vertebrate model for whole-heart in vivo optogenetic studies [10]. Due to their visible transparency, zebrafish larvae, and, to an extent, adult zebrafish, offer a unique advantage for optogenetic applications [63]. This characteristic facilitates efficient optical access for genetic and pharmacological manipulation while enabling the real-time visualization of cardiovascular processes in vivo [60]. The ability to directly observe cardiac morphology, contractility, and electrical activity under physiological conditions makes zebrafish particularly well-suited for optogenetics of heart function. In addition to optical accessibility, zebrafish possess a highly manipulable genome, allowing for the targeted introduction of optogenetic tools and signaling constructs. These features collectively support the precise, spatiotemporal control of cellular activity and reinforce the utility of zebrafish as a powerful model for optogenetic studies in cardiovascular research [64].

### 5.1. Protein-Based Optogenetic Systems for Spatiotemporal Signaling Control

Protein-based optogenetic systems enable the precise, single-cell, real-time modulation of protein function with precise spatiotemporal resolution [65], making them suitable for in vivo studies [15]. In the cardiovascular field, optogenetic tools can be applied to both activate and silence cardiac activity, providing bidirectional control over cardiac activity [66]. Originally developed for neuroscience applications, optogenetics has rapidly expanded into cardiovascular research, where it is now applied to study and modulate cardiac physiology and disease [67,68]. Among these systems, light-inducible dimerization platforms such as iLID are widely used. iLid is based on the AsLOV2 domain, which undergoes a conformational change upon light exposure to expose a peptide motif, thereby enabling reversible protein–protein interactions [65,69,70]. Importantly, the AsLOV2 photoreceptor can also be engineered to regulate cellular activity through mechanisms beyond protein recruitment, including the direct control of ion channel gating [71,72]. In zebrafish embryos, an LOV2-based optogenetic strategy has successfully been applied to achieve the precise spatiotemporal control of intracellular signaling, highlighting the suitability of protein-based optogenetic systems such as iLID for probing dynamic signaling processes in intact tissues [73]. In zebrafish, the microinjection of RNA or DNA into fertilized eggs provides a reliable strategy for the widespread expression of optogenetic constructs [64], while molecular cloning approaches enable the pathway-specific optogenetic control of intracellular signaling [74,75]. Recently, the zHORSE optogenetic zebrafish strain has been developed to allow a spatiotemporal and single-cell level of gene expression during development [69].

### 5.2. Opsin-Based Optogenetic Modulation of Cardiac Electrophysiology

**Figure 5:**
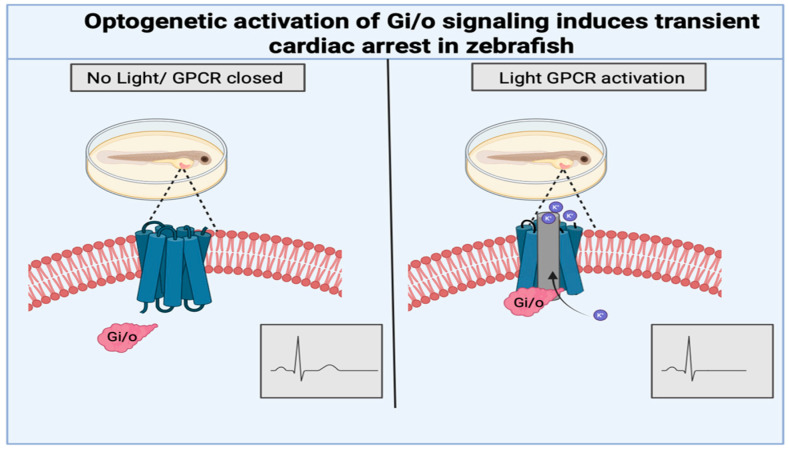
GPCR-based optogenetic control of cardiac pacemaking in zebrafish. Light-activated GPCR opsins expressed in zebrafish cardiomyocytes enable optical activation of inhibitory Gi/o signaling. Illumination triggers downstream potassium channel activity, leading to membrane hyperpolarization and reversible suppression of cardiac electrical activity. Created in BioRender. Avila, V. (2026) https://BioRender.com/r2o839u.

**Figure 6:**
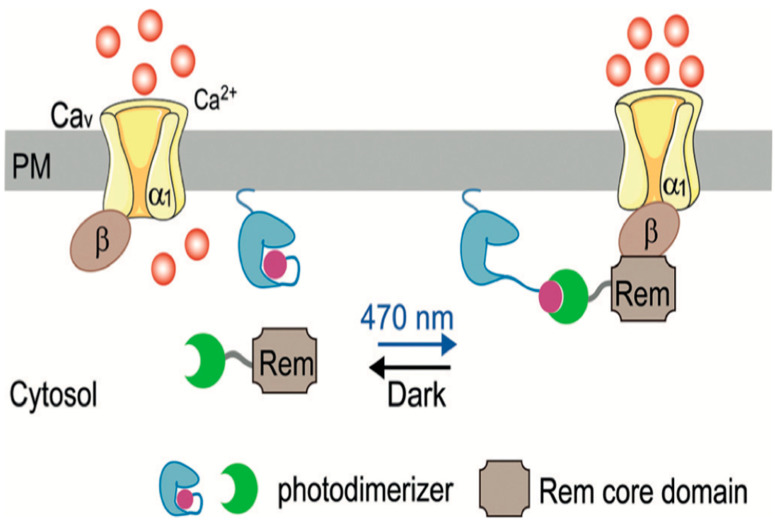
Engineered optoRGK for optical regulation of Ca^2+^ channel activity. In this system, the RGK (yellow) protein Rem inhibits voltage-gated Ca^2+^ channels by associating with the Ca_v_ β subunit (red) at the plasma membrane under dark conditions. Blue light stimulation (470 nm) induces photodimerization-mediated sequestration of Rem away from the channel complex, thereby relieving channel inhibition and restoring Ca^2+^ influx. This approach enables reversible and precise optical control of calcium signaling [86].

In contrast to protein-based optogenetic systems, opsin-based tools directly modulate membrane excitability through light-gated ion channels [76]. This microbial-derived photoreceptor enables the light-dependent control of ion flux across the plasma membrane, allowing the precise optical manipulation of cardiac electrophysiology [75]. Opsins have been adapted to regulate cardiac electrical activity by controlling Na^+^, K^+^, and Ca^2+^ conductance, which together govern action potential initiation and propagation in cardiomyocytes [77,78,79,80]. Because cardiac rhythm depends on tightly regulated electrical timing, the optogenetic modulation of ion flux provides a powerful means to either stimulate or silence cardiac activity [8]. In addition to direct ion channel control, opsin-based strategies have been developed to modulate cardiac function through GPCR signaling pathways; GPCRs are known to hyperpolarize cells by increasing K^+^ efflux. Tagging signaling proteins like GPCRs with rhodopsins enables optogenetic manipulation in zebrafish cardiomyocytes [81]. For example, rhodopsin-based GPCR actuators have been used to optically activate inhibitory Gi signaling in cardiomyocytes, allowing for the rapid and reversible modulation of pacemaking and temporal precision that complements optogenetic approaches [82]. Most recently, the use of the zebrafish expression of Gi/o-coupled rhodopsin in cardiomyocytes allowed the light-dependent activation of inhibitory Gi/o signaling, which engages inward-rectifier K^+^ channels and induces transient cardiac arrest [81] (Figure 5). Blue-light-induced K^+^ gated channels (BLINK1, BLINK2) engineered using LOV-Jα photoreceptor modules have been used to induce hyperpolarization in cardiomyocytes [69,71]. Calcium signaling, another key regulator for cardiac contractility, has also been targeted using optogenetic approaches. In zebrafish, early in vivo studies established that the optogenetic manipulation of cardiomyocytes excitability directly alters excitation, highlighting the feasibility of the optical control of calcium-dependent cardiac function [83]. LOV2-based systems have been developed to regulate the subcellular localization of Ca^2+^ channel inhibitors such as the Ras-like GTPase REM [84]. Similarly, optically dimerized negative regulators of Ca^2+^ channels (opto-RGK) enable the reversible modulation of Ca^2+^ influx, restoring channel activity under dark conditions (Figure 6) [67,85]. Although these Ca^2+^ regulatory systems were characterized in stem cell-derived cardiomyocytes, their modular design and reliance on conserved cardiomyocyte signaling pathways make them directly compatible with zebrafish cardiomyocytes [83].

### 5.3. Optical Pacemakers and Antiarrhythmic Applications

Optogenetic strategies are increasingly explored as alternatives to conventional electrical approaches for controlling cardiac rhythm and terminating arrhythmia. Electrophysiological disorders associated with ischemic heart disease, myocarditis, myocardial infarction, genetic abnormalities, and congenital defects often result in arrhythmias [75,80]. In cardiomyocytes, voltage-gated ion channels mediate depolarization and repolarization events that generate action potentials [79], and the optogenetic control of these channels provides a novel means of rhythm modulation. The optogenetic pacing of the heart has been achieved using embryonic cardiomyocytes, a specific expression of light-gated ion channels, which has enabled the location and control of pacemaker cells [83]. Subsequently, ligand-gated systems such as the vertebrate cation channel zTrpa1b paired with photo-activatable compounds have enabled the high-conductance, millisecond-scale pacing of zebrafish cardiomyocytes in vivo, offering tunable and precise rhythm modulation [87]. Notably, the optogenetic depolarization of light-gated channels has been shown to terminate ventricular tachycardia with high efficiency [86], highlighting the potential of optical pacemakers as refined, pain-free antiarrhythmic therapies. Compared with electrical pacemakers, optical pacing offers superior spatial precision and cell-type specificity, reducing the off-target stimulation of skeletal muscle, diaphragm, and vocal cords that commonly accompanies electrical defibrillation [80]. By enabling the selective depolarization or hyperpolarization of cardiomyocytes, optogenetic actuators may overcome key limitations of existing therapeutic strategies for cardiac rhythm control [88].

### 5.4. Optical Vagal Stimulation for Heart Regeneration

Following myocardial infarction, large numbers of cardiomyocytes are lost, and, because adult mammalian cardiomyocytes exhibit minimal regenerative capacity, injured myocardium is typically repaired by fibrotic scar tissue rather than functional muscle replacement [89,90,91]. In contrast, zebrafish retain robust regenerative capacity, highlighting that cardiac regeneration is biologically feasible. Vagus nerve stimulation has been successfully applied as a neuromodulator strategy to reduce heart rate and promote myocardial repair after injury in the mammalian system [92,93]. Increasing evidence suggests that parasympathetic activation also influences cardiomyocyte biology and the post-injury process [94]. More recently, optogenetic approaches have enabled the cell-type selective stimulation of the cardiac vagus nerve, including targeted activation at the level of vagal ganglia, providing a precise neuro-cardiac communication without off-target stimulation [93]. Notably, optogenetic vagal activation has been reported to increase cardiomyocyte proliferation markers, improve ventricular remodeling, and enhance regenerative repair following myocardial infarction [95]. Nevertheless, zebrafish optical accessibility and regenerative capacity support their use as a tractable vertebrate model to implement vagal activation with the help of optogenetic tools and offer opportunities to bridge neuromodulation and regenerative biology [90,96].

## 6. Nanoparticle-Enabled Optogenetics: A Synergistic Approach

**Figure 7:**
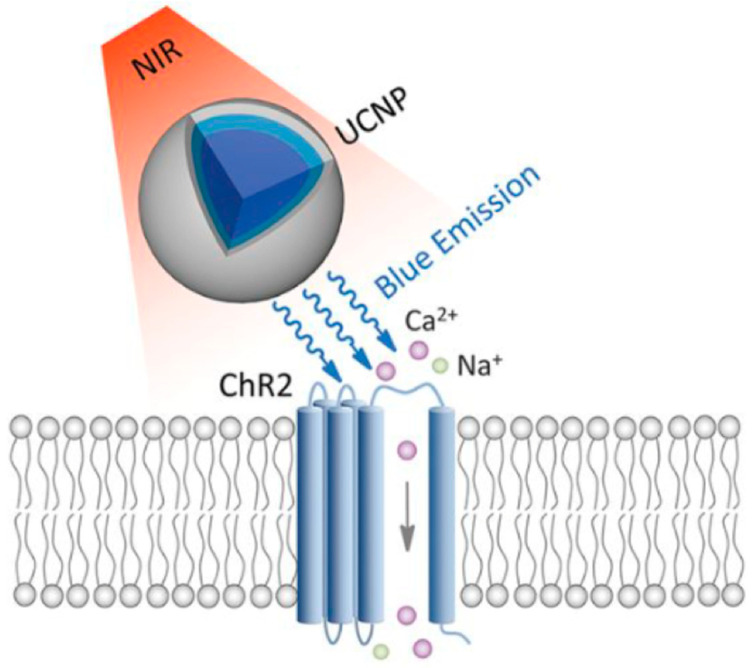
Schematic of UCNP-assisted optogenetic stimulation. UCNPs convert NIR light into blue emission that activates ChR2 channels, inducing Na^+^ and Ca^2+^ influx and membrane depolarization [104].

**Figure 8:**
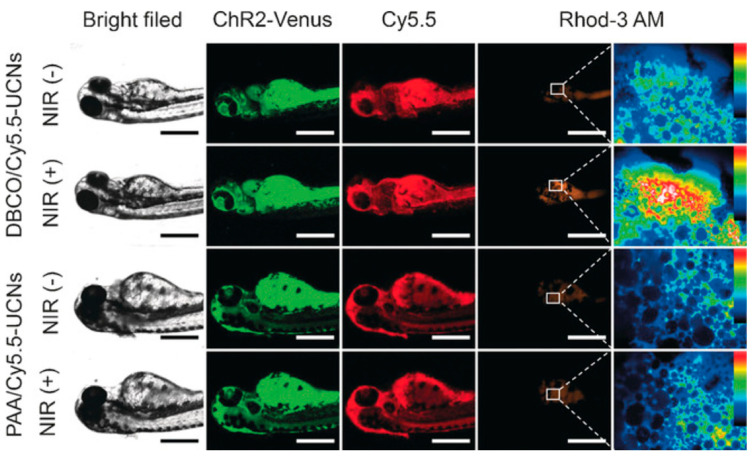
Fluorescence images of zebrafish larvae demonstrating site-specific localization of upconversion nanoparticles and near-infrared (NIR)-mediated activation of optogenetic channels. NIR illumination induces localized calcium signaling only in larvae treated with surface-localized nanoparticles, whereas control conditions show minimal response. This illustrates nanoparticle-enabled optical transduction as a proof-of-concept strategy for remote modulation of ion channel activity in vivo [103].

Nanotechnology and optogenetics can complement one another by addressing technical limitations inherent to each other’s approaches. While optogenetics enables the precise spatiotemporal control of genetically modified cells using light [97], its broader application is limited by light penetration into deep tissues and the challenge associated with target gene delivery [98]. Lanthanide-doped upconversion nanoparticles (UCNPs) act as optical transducers, capable of converting tissue penetrant near-infrared (NIR) light into visible wavelengths, capable of activating opsins [67]. When NIR light is applied externally, UCNPs emit blue or green light locally within cardiac tissue, enabling wireless and non-invasive optogenetic stimulation without implanted optical fibers or invasive surgery [99,100,101,102] (Figure 7). Following near-infrared light exposure, zebrafish larvae with surface-localized upconversion nanoparticles exhibited increased ion influx, whereas control larvae showed minimal response [103]. This demonstrates that nanoparticle localization enables the optical modulation of ion channel activity in vivo (Figure 8). However, these approaches remain largely proof-of-concept and face important limitations. Additionally, zebrafish provide a particularly suitable platform for exploring and refining nanoparticle and optogenetic complementary approaches, given their optical transparency, external development, and compatibility with embryo microinjection, nanoparticle delivery, and bath-based exposure methods. In addition to optical transduction, nanoparticles can also function as non-viral carriers for delivering optogenetic genes to cardiomyocytes, reducing reliance on viral vectors and enabling delivery efficiency, cardiac targeting, and functional outcomes [104,105].

## 7. Perspective and Outlook

The integration of nanoparticle-based technologies with optogenetic tools has positioned zebrafish as a uniquely powerful platform for cardiovascular research. By enabling the simultaneous control and visualization of molecular signaling, electrophysiology, and tissue-level function, these approaches bridge mechanistic discovery and translational innovation. Continued advances in biomimetic nanocarriers, stimulus-responsive systems, and non-invasive optogenetic strategies are expected to further expand the utility of zebrafish models in precision cardiovascular studies.

## Figures and Tables

**Table 1:** Comparative summary of nanoparticle-based and optogenetic approaches used in zebrafish cardiovascular research. The table highlights key advantages and limitations of commonly used nanoparticle platforms, optogenetic tools, and nanoparticle-enabled optogenetic strategies.

Platform	Key Advantages	Key Limitations
PLGA polymeric NPs	Tunable degradation; sustained release; strong clinical precedent	Limited intrinsic targeting; uptake reduced by PEGylation
PEGylated NPs	Extended circulation; murine-like pharmacokinetics	Reduced cellular uptake; “stealth” limits myocardial internalization
Chitosan NPs	High nucleic acid loading; efficient endosomal escape	Cationic toxicity; non-specific uptake; mitochondrial stress
Lipid-based NPs (liposomes, LNPs)	High biocompatibility; efficient mRNA delivery	Short-lived expression; leakage under flow
Surface-functionalized NPs	Ligand-directed cardiac/endothelial targeting	Ligand density optimization is complex
Biomimetic membrane-coated NPs	Superior injury-site specificity; immune evasion	Complex fabrication; reproducibility challenges
Stimuli-responsive NPs	Disease-activated release; high spatial precision	Dependence on pathological thresholds
Carbon-based NPs	Sensitive models for inflammation and toxicity	Intrinsic cardiotoxicity
Iron oxide / inorganic NPs	Dual imaging–functional readouts	ROS imbalance at higher doses
Protein-based optogenetic systems (LOV/iLID, signaling control)	Precise spatiotemporal control of signaling pathways; reversible and cell-specific	Requires genetic expression; limited by light penetration.
Opsin-based optogenetics (ion channels, optical pacing)	Direct, strong physiological readout; suited for electrophysiology; works well in zebrafish larvae	Requires strong opsin expression; limited light penetration and off target stimulation.
Nanoparticle-enabled Optogenetics	Potential for cardiac targeting via ligands; enables combined imaging + actuation strategies	Low delivery efficiency
Upconversion NPs (optogenetics)	Can enable remote activation without direct blue light exposure at the target region	Requires careful alignment of UCNP emission with opsin absorption

## Data Availability

No new data were created or analyzed in this study.
